# Day-case gastrostomy insertion in children: an achievable reality

**DOI:** 10.1007/s00383-024-05929-0

**Published:** 2025-01-24

**Authors:** David Thompson, Maddie Allam, Karen Dick, Jo Leigh, Rhoda Taylor, Charlie Keys, Lara Kitteringham, Ori Ron, Michael Stanton, Francesca Stedman, Nigel J. Hall

**Affiliations:** 1 Department of Paediatric Surgery and Urology, Southampton Children’s Hospital, Southampton, UK; 2 University Surgery Unit, University of Southampton, Southampton, UK

**Keywords:** Gastrostomy, Day case surgery, Paediatric gastrostomy, Gastrostomy insertion

## Abstract

**Purpose:**

Recent efforts have sought to streamline gastrostomy insertion care, particularly length of stay (LOS). We report our initial experience with day-case gastrostomy (DCG) insertion.

**Method:**

Retrospective review (April 2018–2024) of all primary gastrostomy insertions. Patients discharged the same day as the procedure were defined as DCG. Demographic, operative, and clinical data were recorded. All cases were treated according to a standardized feeding pathway.

**Results:**

Of 432 gastrostomies formed, 15 were DCG; median age 3.5 (0.7–16.9) years, LOS 12 h (9–15 h). The most common indication was nutritional supplementation (*n* = 9). Gastrostomy technique was single-stage percutaneous rapid insertion of gastrostomy button (SPRING *n* = 5) or percutaneous endoscopic gastrostomy (PEG *n* = 10). Prior to insertion, 6/15 DCG were established on nasogastric (NG) feeding, 8 did not use NG feeding, and 1 had occasional NG feeds. The majority (13/15) were performed on morning operating lists. There were 4 minor complications; 2 required readmission.

**Conclusion:**

DCG in selected cases is feasible and safe. Most cases were performed on morning operating list, but fewer than half had prior experience of nasogastric tube feeding. We suggest additional pathway modifications to improve DCG uptake.

## Introduction

Gastrostomy use for both feeding and administration of medications forms a cornerstone in the management of many children with both surgical and medical diagnoses. Gastrostomy insertion can be via a range of methods, including percutaneous endoscopic gastrostomy insertion (PEG), laparoscopy-assisted gastrostomy insertion, and open gastrostomy.

The insertion of a new gastrostomy device, by any method, has traditionally involved one or two nights stay in hospital, during which the child’s feed is increased and parents are educated in the use of the gastrostomy device [1, 2]. In recent years, increasing evidence has shown that new gastrostomy devices can be used safely soon after insertion. Early feeding combined with adoption of a protocolised post-insertion pathway has been shown to reduce length of stay following gastrostomy insertion [3, 4], such that in some units, including ours, a stay of one night (as opposed to longer) is now considered routine. This reduction in length of stay has benefits for patients, families, and the health service.

Although day-case surgery is common in paediatric surgery [5], some procedures that are routinely performed on a day-case basis in adults continue to mandate overnight hospital admission in children. Furthermore, there is a wide variation in post-procedure length of stay following many paediatric surgical procedures [6]. Although day-case surgery can bring its own challenges, such as expectation of parents, single day travel for families to tertiary-care hospitals, post-operative pain, and nausea management [7, 8], it also has many benefits including reduced hospital cost, reduced risk of hospital acquired infections, improved convenience for the patient’s family, and improved inpatient bed availability [9].

At our institution, we realised that with optimisation of parent and carer education, early post-operative feeding and enhanced surgical recovery, day-case surgery for gastrostomy insertion could be achievable and safe. Here, we describe our early experience of day-case gastrostomy cases and aim to demonstrate its feasibility in paediatric patients.

## Methods

We performed a retrospective case note review of all primary gastrostomy insertions at our institution over a 6 year period (April 2018–April 2024). Patient demographics and clinical data were retrieved, including age, sex, co-morbidities, indication for gastrostomy, pre-operative nasogastric tube (NGT) use, operative technique, and intra-operative or post-operative complications. Patient length of stay was calculated for each case through reviewing patient admission and discharge times.

From this, we identified two groups of patients—true day-case gastrostomy (DCG) cases, defined as patients who were discharged on the same day as admission for the procedure, and a second group of patients whose total stay was < 24 h but did spend one night in hospital. We report detail of these two groups descriptively. All data are presented as median (range) unless stated otherwise.

During the study period, our department utilised an early feeding pathway for all gastrostomy insertions as previously reported [3]. The surgical technique of insertion is not standardised but is based on surgeon and patient preference alongside relevant surgical factors, including weight, co-morbidities, and prior surgical history. The most frequently used surgical approaches are single-stage percutaneous rapid insertion of gastrostomy button (SPRING) or classic percutaneous endoscopic gastrostomy (PEG), both of which are performed with or without laparoscopic assistance.

### Pathway

Prior to the day of the operation, all of our patients’ parents or carers meet with either a paediatric surgeon or the Paediatric Surgical Nurse Specialist. During this clinic appointment, gastrostomy device options are discussed and the patient is given access to both patient information leaflets and video information.

Patients are admitted on the day of surgery, transferred to the operating room and after the procedure are cared for on the inpatient paediatric surgery ward. All procedures are performed under general anaesthesia. During this post-operative period, parents are provided with further information on how to care for the gastrostomy tube and their competency in a range of gastrostomy related tasks is assessed (appendix 1, 2). Criteria for discharge are: 1. tolerating two full volume feeds, if tube is required for nutritional support; 2. patient has a normal temperature; 3. a consistent score of 0 on the child’s PEWS assessment; 4. insertion site is dry and clean; 5. passing urine; 6. pain free or adequately controlled on appropriate analgesia; 7. achieving tube competencies. Further education regarding tube use is continued by the community nursing team. Discharge information includes daytime and emergency contact details of the paediatric surgery team.

## Results

During the study period, 432 new gastrostomies were inserted. Fifteen (3.5%) of these were DCG. Median DCG patient age was 5.6 (0.8–16.9) years. Median length of stay for DCG was 12 h (9–15 h). Thirteen cases were performed on a morning operating list, two on an afternoon list. A further 9 cases had an overnight stay but were discharged within 24 h of recovery from anaesthesia.

For the DCG group, indication for gastrostomy insertion was: nutritional supplementation (*n* = 9), unsafe swallow (*n* = 3), medication administration (*n* = 2), and prophylactically prior to proton radiotherapy (*n* = 1).

Gastrostomy technique was either: SPRING in 5 (4 laparoscopy-assisted) or PEG in 10 (6 laparoscopy-assisted).

Prior to insertion, 6 of 15 DCG cases were established on NGT feeding, and one patient had occasional use of an NGT but had a sibling that was already established on gastrostomy feeding. The remaining 8 patients did not have any experience of tube feeding or regularly use a NGT for medications.

For DCG cases, there were no intra-operative complications and none had a return to theatre. Four of the 15 cases experienced a minor complication following DCG, two of which required admission to hospital. One was admitted with a gastrostomy site infection, managed with intravenous antibiotic. A second patient was admitted to their local hospital with concern of bleeding from a laparoscopic port site; this was managed locally with vitamin K and oral antibiotics. One patient was reviewed by our specialist nursing team with parental concerns regarding the gastrostomy fixation site. One patient was reviewed in the community with a possible gastrostomy site infection and was managed at home with oral antibiotics. None of these events occurred within the first 24 h following the procedure.

For the nine cases whose stay was less than 24 h but required one overnight night stay, patient age was median 2.7 (0.7–13.3) years and total length of stay was 23 h (18–24 h). Indication for gastrostomy was: nutritional supplementation (*n* = 8) and unsafe swallow (*n* = 1). Operative technique was: laparoscopy-assisted SPRING (*n* = 3) or PEG (*n* = 6, 1 laparoscopy-assisted), one case had an old gastrostomy site closed at the same time as a new PEG inserted. Eight of these nine cases were performed on an afternoon operating list.

## Discussion

Here, we report our initial experience of DCG, aiming to highlight that in selected cases, it is achievable and safe. Although we report only 15 cases performed as a day-case, we believe that this represents significant progress in the peri-operative care of this common paediatric surgical procedure. By reviewing our experience of treating these children, we have identified opportunities to refine our pathways aiming to offer this capability to more families in the future, whilst recognising this will not be suitable for all patients and their families or carers. By sharing our early experience, we hope that others may feel similarly inspired to push the boundaries in relation to planned post-operative length of stay.

Our achievement of undertaking gastrostomy insertion as a day-case procedure is a natural progression of our intention over some years to refine post-operative care pathways. Having previously demonstrated that an early post-operative feeding pathway is safe and is associated with reduced post-operative length of stay compared to a non-protocolised approach [3], we found that some children were ready for discharge the same day and discharged them. Although, at present, the total number who were completed as a DCG is relatively small, we believe that it is likely that this group could be expanded relatively easily in the future having demonstrated its feasibility here. To support this belief, we have already identified a similar number of cases in whom length of stay was less than 24 h. It is likely that with minimal modifications to our processes, these could also be achieved as a day-case.

An analysis to determine if there are patient factors that can predict suitability for DCG is beyond the scope of this report but will form part of our future investigations. Clearly, there are a range of non-modifiable patient factors why an overnight stay may be required such as co-morbidities and anaesthetic considerations but there may be specific patient groups who can be identified as highly suitable (or not) for DCG.

Enhanced recovery after surgery (ERAS) pathways are widely used in many surgical disciplines but relatively under-utilised in paediatric surgery [10, 11]. We acknowledge that many of the principles we have employed are similar to those commonly found in ERAS pathways and note that others have recently reported the establishment of an ERAS pathway in children undergoing gastrostomy insertion. They have not reported any cases performed as a day-case. Future work will involve adapting our pathway further to ensure that all aspects of ERAS pathways (pre-operative, intra-operative, and post-operative) are incorporated and adequately optimised [10].

We also note previous reports focussed on early post-operative feeding, in whom inpatient stay of less than 24 h was achieved in some cases with similar positive outcomes to our cohort [1, 2, 12, 13]. As far as we are aware, there is just one previous report of true DCG. Dekonenko et al. reported their early experience with DCG. Patients were eligible if they met three criteria: 1) family were comfortable with DCG and patient had no major co-morbidities, 2) gastrostomy was the only procedure, and 3) the family had pre-operative education. With this, they achieved a DCG rate of 82% (*n* = 51) from 62 eligible patients [14].

Interestingly, we found that 53% (8/15) DCG patients had not had prior NGT use before their gastrostomy insertion. Prior to analysing our data, we had hypothesised that parent or carer experience and familiarity with NGT feeding would be essential to achieve DCG. This does not appear to be the case and perhaps points to additional factors that are important in successfully discharging a gastrostomy as a day-case.

In our practice, families will have met with a specialist nurse prior to their surgery day to discuss gastrostomy options and given both literature and videos to review. This aims to familiarise families with the gastrostomy device; however, formal education regarding gastrostomy use does not start until after the procedure. We note that the majority of DCG cases (13/15; 87%) were performed on a morning operating list likely allowing longer time the same day for education to occur. Preferentially listing patients on a morning operating list would be a logical step if aiming for same-day discharge.

Moving forwards, we consider a number of features will be important as we shift our focus towards achieving higher numbers of DCG:1) setting the expectation with parents and staff from an early stage that the procedure can be completed as a day-case, 2) optimising pre- and post-operative parent education, starting education of gastrostomy use prior to patient returning from theatre, 3) ensuring that any equipment needs are identified and fulfilled in advance, so that is this not a rate-limiting step when it comes to discharge, and 4) listing patients on a morning operating list.

Given the frequency of gastrostomy in paediatric surgical practice, and the growing prevalence of gastrostomies in the paediatric population [15], we believe that there is significant opportunity to bring benefit to patients and the healthcare system by continuing to advance our practice in this field.

Limitations of this study include the retrospective nature of the data collection and the relatively low number of cases. As a department, we are early in our journey of DCG; our aim at this early stage has been to report its feasibility and safety. As we gain greater experience with patient selection and refine our pathways, we hope to report a larger experience and share our learning.

## Conclusion

DCG is feasible and safe in selected cases. Most cases were performed on morning operating list, but fewer than half had prior experience of nasogastric tube feeding. We suggest these and additional considerations to refine our pathway aiming to achieve a greater proportion of same-day discharge cases.

## Data Availability

No datasets were generated or analysed during the current study.
